# (*E*)-3-(2-Nitro­phen­yl)-1-{1-phenyl­sulfonyl-2-[(phenyl­sulfon­yl)meth­yl]-1*H*-indol-3-yl}prop-2-en-1-one

**DOI:** 10.1107/S1600536811051026

**Published:** 2011-12-03

**Authors:** S. Karthikeyan, K. Sethusankar, Ganesan Gobi Rajeswaran, Arasambattu K. Mohanakrishnan

**Affiliations:** a Department of Physics, RKM Vivekananda College (Autonomous), Chennai 600 004, India; b Department of Organic Chemistry, University of Madras, Maraimalai Campus, Chennai 600 025, India

## Abstract

In the title compound, C_30_H_22_N_2_O_7_S_2_, the configuration about the propene C=C bond is *E*. The indole unit is essentially planar, with a maximum deviation of 0.031 (3) Å. The dihedral angle between the planes of the phenyl rings of the two phenyl­sulfonyl groups is 80.95 (19)°. The central prop-2-ene-1-one group is oriented at a dihedral angle of 44.26 (11)° with respect to the nitro­phenyl ring and at 39.24 (8)° with respect to the indole unit. The S atoms are in a distorted tetra­hedral configuration. In the crystal, mol­ecules are linked into centrosymmetric dimers *via* pairs of C—H⋯O hydrogen bonds with an *R*_2_^2^(24) graph-set motif. The crystal structure is stabilized by further C—H⋯O inter­actions. Short intra­molecular C—H⋯O contacts result in several S(6) rings.

## Related literature

For the biological activity of sulfonamides and their substituted derivatives, see: Brown (1971 ▶). For related structures, see: Seetharaman & Rajan (1995 ▶); Varghese *et al.* (1986 ▶). For graph-set motifs, see: Bernstein *et al.* (1995 ▶). 
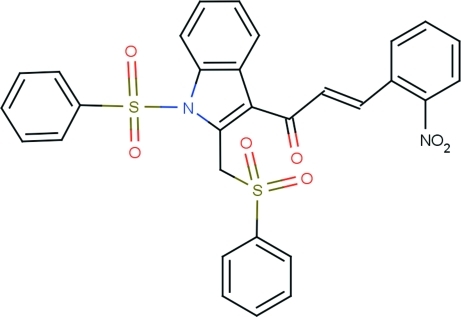


## Experimental

### 

#### Crystal data

C_30_H_22_N_2_O_7_S_2_
*M* *_r_* = 586.62
Monoclinic, 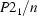

*a* = 7.9905 (2) Å
*b* = 22.2076 (6) Å
*c* = 15.7378 (4) Å
β = 102.913 (2)°
*V* = 2722.04 (12) Å^3^
*Z* = 4
Mo *K*α radiation
μ = 0.25 mm^−1^
*T* = 293 K
0.30 × 0.25 × 0.20 mm

#### Data collection

Bruker Kappa APEXII CCD diffractometer
32862 measured reflections
6823 independent reflections
4782 reflections with *I* > 2σ(*I*)
*R*_int_ = 0.030

#### Refinement

*R*[*F* ^2^ > 2σ(*F* ^2^)] = 0.058
*wR*(*F* ^2^) = 0.186
*S* = 1.03
6823 reflections
370 parameters
H-atom parameters constrained
Δρ_max_ = 1.01 e Å^−3^
Δρ_min_ = −0.36 e Å^−3^

### 

Data collection: *APEX2* (Bruker, 2008 ▶); cell refinement: *SAINT* (Bruker, 2008 ▶); data reduction: *SAINT*; program(s) used to solve structure: *SHELXS97* (Sheldrick, 2008 ▶); program(s) used to refine structure: *SHELXL97* (Sheldrick, 2008 ▶); molecular graphics: *OLEX2* (Dolomanov *et al.*, 2009 ▶) and *Mercury* (Macrae *et al.*, 2008 ▶); software used to prepare material for publication: *SHELXL97* and *PLATON* (Spek, 2009 ▶).

## Supplementary Material

Crystal structure: contains datablock(s) global, I. DOI: 10.1107/S1600536811051026/pv2489sup1.cif

Structure factors: contains datablock(s) I. DOI: 10.1107/S1600536811051026/pv2489Isup2.hkl

Supplementary material file. DOI: 10.1107/S1600536811051026/pv2489Isup3.cml

Additional supplementary materials:  crystallographic information; 3D view; checkCIF report

## Figures and Tables

**Table 1:** Hydrogen-bond geometry (Å, °)

*D*—H⋯*A*	*D*—H	H⋯*A*	*D*⋯*A*	*D*—H⋯*A*
C27—H27⋯O2^i^	0.93	2.56	3.221 (3)	128
C13—H13⋯O1^ii^	0.93	2.58	3.275 (5)	132
C19—H19⋯O4^iii^	0.93	2.58	3.222 (4)	126
C2—H2⋯O4	0.93	2.37	2.946 (4)	120
C9—H9*A*⋯O3	0.97	2.21	2.846 (3)	122
C9—H9*B*⋯O5	0.97	2.37	3.029 (3)	125

Symmetry codes: (i) 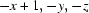; (ii) 
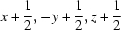
; (iii) 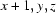.

**Fig. 1.**
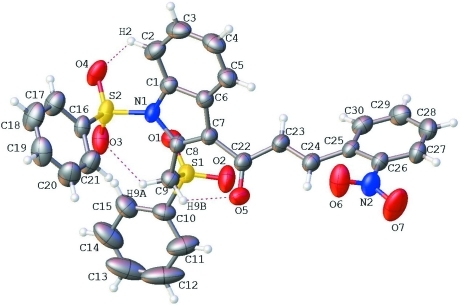
The molecular structure of the title compound with the atom numbering scheme, displacement ellipsoids are drawn at 30% probability level. The intramolecular S(6) ring motiffs have been drawn by dashed lines.

**Fig. 2.**
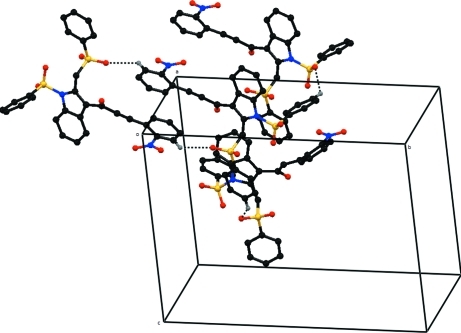
The packing arrangement of the title compound viewed down c axis, showing the formation of centrosymmetric R22(24) dimer. The dashed lines indicate C—H···O intermolecular interactions.

## supplementary crystallographic information

### Comment

Sulfonamides and their substituted derivatives are well known drugs and are commonly used to control diseases caused by bacterial infections (Brown, 1971). Herein we report the synthesis and crystal structure of a novel sulfonamides derivative.

In the title compound (Fig. 1), the indole moiety is essentially planar with a maximum deviation of 0.031 (3)Å for the atom C6. The configuration of the keto group with respect to the olefinic double bond is typically *S-cis*, with O5—C22—C23—C24 torsion angle -18.1 (4)°. The propenone group exhibits an E configuration with respect to the C23═C24 double bond.

The indole moiety, is perpendicular to both the nitro phenyl ring and the phenylsulfonyl ring, bonded to the N atom of the indole ring system with interplanar angles 81.04 (11) and 89.21 (14)°, respectively. The methyl substituted phenylsulfonyl ring is inclined with respect to the indole moiety and the phenylsulfonyl ring, bonded to N atom of the indole ring system at angles of 9.01 (16)° and 80.95 (19)°. The two sulfonyl bound phenyl rings make a dihedral angle of 73.17 (17)° and 52.35 (15)° with the nitro phenyl ring. The nitro-group is inclined at an angle of 27.63 (16)° with the benzene ring, to which it is attached.

The molecular dimensions in the title compound are in excellent agreement with the corresponding molecular dimensions reported in closely related compounds (Varghese *et al.*, 1986; Seetharaman & Rajan, 1995).

The crystal packing is stabilized by intermolecular C—H···O interactions and molecules are stacked along the *a* axis; the molecules are linked into centrosymmetric dimers *via* pairs of C—H···O hydrogen bonds with an *R*^2^_2_(24) graph-set motif (Bernstein, *et al.*, 1995) (Fig. 2). Intramolecular C—H···O hydrogen bonds generate S(6) ring motifs.

### Experimental

To a solution of (*E*)-1-(2-(bromomethyl)-1-phenylsulfonyl-indol-3-yl)-3-(2- nitrophenyl)prop-2-en-1-one (0.5 g, 0.95 mmol) in dimethylformamide (5 ml), sodium phenylsulfinate (0.18 g, 1.1 mmol) was added and stirred for 5 h at room temperature. After completion of the reaction (monitored by TLC), the mixture was poured over crushed ice (100 g). The solid (0.5 g, 90%) formed was filtered and recrystallized from MeOH to afford the title compund as colorless crystals.

### Refinement

The hydrogen atoms were placed in calculated positions with C—H = 0.93 and 0.97Å for aryl and methylene H-atoms, respectively, and refined in the riding model with *U*_iso_(H) = 1.2*U*_eq_(C).

### Crystal data

**Table:**

C_30_H_22_N_2_O_7_S_2_	*F*(000) = 1216
*M**_r_* = 586.62	*D*_x_ = 1.431 Mg m^−^^3^
Monoclinic, *P*2_1_/*n*	Mo *K*α radiation, λ = 0.71073 Å
Hall symbol: -p 2yn	Cell parameters from 6823 reflections
*a* = 7.9905 (2) Å	θ = 1.6–28.5°
*b* = 22.2076 (6) Å	µ = 0.25 mm^−^^1^
*c* = 15.7378 (4) Å	*T* = 293 K
β = 102.913 (2)°	Block, colourless
*V* = 2722.04 (12) Å^3^	0.30 × 0.25 × 0.20 mm
*Z* = 4	

### Data collection

**Table:**

Bruker Kappa APEXII CCD diffractometer	4782 reflections with *I* > 2σ(*I*)
Radiation source: fine-focus sealed tube	*R*_int_ = 0.030
graphite	θ_max_ = 28.5°, θ_min_ = 1.6°
ω and φ scans	*h* = −10→10
32862 measured reflections	*k* = −29→29
6823 independent reflections	*l* = −20→20

### Refinement

**Table:**

Refinement on *F*^2^	Primary atom site location: structure-invariant direct methods
Least-squares matrix: full	Secondary atom site location: difference Fourier map
*R*[*F*^2^ > 2σ(*F*^2^)] = 0.058	Hydrogen site location: inferred from neighbouring sites
*wR*(*F*^2^) = 0.186	H-atom parameters constrained
*S* = 1.03	*w* = 1/[σ^2^(*F*_o_^2^) + (0.0995*P*)^2^ + 1.3462*P*] where *P* = (*F*_o_^2^ + 2*F*_c_^2^)/3
6823 reflections	(Δ/σ)_max_ < 0.001
370 parameters	Δρ_max_ = 1.01 e Å^−^^3^
0 restraints	Δρ_min_ = −0.36 e Å^−^^3^

### Special details

**Table:**

Geometry. All s.u.'s (except the s.u.'s in the dihedral angle between two l.s. planes) are estimated using the full covariance matrix. The cell s.u.'s are taken into account individually in the estimation of s.u.'s in distances, angles and torsion angles; correlations between s.u.'s in cell parameters are only used when they are defined by crystal symmetry. An approximate (isotropic) treatment of cell s.u.'s is used for estimating s.u.'s involving l.s. planes.
Refinement. Refinement of F^2^ against ALL reflections. The weighted R-factor wR and goodness of fit S are based on F^2^, conventional R-factors R are based on F, with F set to zero for negative F^2^. The threshold expression of F^2^ > 2sigma(F^2^) is used only for calculating R-factors(gt) etc. and is not relevant to the choice of reflections for refinement. R-factors based on F^2^ are statistically about twice as large as those based on F, and R- factors based on ALL data will be even larger.

### Fractional atomic coordinates and isotropic or equivalent isotropic displacement parameters (Å^2^)

**Table:**

	*x*	*y*	*z*	*U*_iso_*/*U*_eq_	
C1	0.5998 (3)	0.34651 (11)	−0.01613 (16)	0.0425 (5)	
C2	0.5611 (4)	0.39178 (13)	−0.0784 (2)	0.0578 (7)	
H2	0.5405	0.4312	−0.0634	0.069*	
C3	0.5547 (4)	0.37511 (15)	−0.1644 (2)	0.0664 (8)	
H3	0.5277	0.4041	−0.2080	0.080*	
C4	0.5871 (4)	0.31678 (16)	−0.18715 (19)	0.0632 (8)	
H4	0.5831	0.3076	−0.2452	0.076*	
C5	0.6251 (4)	0.27239 (13)	−0.12536 (17)	0.0542 (6)	
H5	0.6473	0.2333	−0.1410	0.065*	
C6	0.6299 (3)	0.28697 (11)	−0.03834 (15)	0.0424 (5)	
C7	0.6711 (3)	0.25300 (10)	0.04173 (15)	0.0406 (5)	
C8	0.6668 (3)	0.29053 (10)	0.10888 (15)	0.0404 (5)	
C9	0.6959 (3)	0.27418 (12)	0.20279 (16)	0.0470 (6)	
H9A	0.7441	0.3086	0.2378	0.056*	
H9B	0.7789	0.2417	0.2150	0.056*	
C10	0.5613 (4)	0.25333 (14)	0.34824 (17)	0.0547 (7)	
C11	0.6166 (5)	0.2026 (2)	0.3950 (3)	0.0902 (12)	
H11	0.6241	0.1659	0.3678	0.108*	
C12	0.6625 (6)	0.2085 (3)	0.4877 (3)	0.127 (2)	
H12	0.7036	0.1755	0.5224	0.152*	
C13	0.6456 (6)	0.2634 (4)	0.5250 (3)	0.124 (2)	
H13	0.6718	0.2664	0.5855	0.149*	
C14	0.5929 (5)	0.3125 (3)	0.4777 (2)	0.0962 (14)	
H14	0.5855	0.3491	0.5051	0.115*	
C15	0.5497 (4)	0.30874 (17)	0.38864 (19)	0.0664 (8)	
H15	0.5129	0.3428	0.3553	0.080*	
C16	0.8420 (3)	0.43956 (11)	0.13095 (17)	0.0469 (6)	
C17	0.8594 (4)	0.49609 (13)	0.0994 (2)	0.0638 (8)	
H17	0.7633	0.5187	0.0740	0.077*	
C18	1.0234 (5)	0.51890 (15)	0.1062 (3)	0.0761 (10)	
H18	1.0383	0.5576	0.0866	0.091*	
C19	1.1633 (4)	0.48428 (16)	0.1420 (2)	0.0733 (9)	
H19	1.2729	0.4998	0.1461	0.088*	
C20	1.1451 (4)	0.42794 (16)	0.1714 (3)	0.0743 (9)	
H20	1.2414	0.4047	0.1944	0.089*	
C21	0.9823 (4)	0.40518 (14)	0.1672 (2)	0.0672 (8)	
H21	0.9682	0.3670	0.1887	0.081*	
C22	0.7121 (3)	0.18772 (11)	0.05105 (16)	0.0459 (5)	
C23	0.6136 (4)	0.14762 (11)	−0.01649 (17)	0.0487 (6)	
H23	0.5117	0.1610	−0.0524	0.058*	
C24	0.6704 (3)	0.09237 (11)	−0.02613 (16)	0.0463 (6)	
H24	0.7785	0.0822	0.0067	0.056*	
C25	0.5766 (3)	0.04638 (10)	−0.08421 (15)	0.0421 (5)	
C26	0.6559 (3)	−0.00166 (10)	−0.11791 (16)	0.0438 (5)	
C27	0.5667 (4)	−0.04876 (12)	−0.16277 (19)	0.0543 (6)	
H27	0.6252	−0.0803	−0.1821	0.065*	
C28	0.3914 (4)	−0.04922 (13)	−0.1790 (2)	0.0611 (7)	
H28	0.3296	−0.0809	−0.2096	0.073*	
C29	0.3070 (4)	−0.00207 (13)	−0.1496 (2)	0.0597 (7)	
H29	0.1876	−0.0015	−0.1618	0.072*	
C30	0.3974 (4)	0.04393 (12)	−0.10241 (18)	0.0531 (6)	
H30	0.3376	0.0745	−0.0818	0.064*	
N1	0.6179 (3)	0.34835 (8)	0.07519 (13)	0.0428 (4)	
N2	0.8416 (3)	−0.00196 (11)	−0.10846 (18)	0.0606 (6)	
O1	0.3735 (3)	0.29516 (9)	0.20506 (12)	0.0577 (5)	
O2	0.4684 (3)	0.19066 (9)	0.20544 (16)	0.0751 (7)	
O3	0.6243 (3)	0.39890 (9)	0.21694 (14)	0.0672 (6)	
O4	0.5114 (3)	0.45290 (8)	0.08011 (16)	0.0676 (6)	
O5	0.8204 (3)	0.16859 (9)	0.11194 (12)	0.0604 (5)	
O6	0.9183 (3)	0.04543 (11)	−0.1026 (2)	0.0877 (8)	
O7	0.9119 (3)	−0.05005 (12)	−0.1109 (3)	0.1135 (12)	
S1	0.50305 (9)	0.25119 (3)	0.23386 (4)	0.04646 (18)	
S2	0.63430 (9)	0.41325 (3)	0.13067 (5)	0.0503 (2)	

### Atomic displacement parameters (Å^2^)

**Table:**

	*U*^11^	*U*^22^	*U*^33^	*U*^12^	*U*^13^	*U*^23^
C1	0.0416 (12)	0.0404 (12)	0.0460 (13)	−0.0016 (9)	0.0107 (10)	0.0032 (10)
C2	0.0551 (15)	0.0460 (14)	0.0715 (19)	0.0000 (12)	0.0126 (13)	0.0161 (13)
C3	0.0644 (18)	0.072 (2)	0.0571 (17)	−0.0060 (15)	0.0018 (14)	0.0269 (15)
C4	0.0643 (18)	0.080 (2)	0.0420 (14)	−0.0120 (16)	0.0059 (12)	0.0064 (14)
C5	0.0610 (16)	0.0578 (16)	0.0434 (14)	−0.0039 (13)	0.0105 (12)	−0.0020 (11)
C6	0.0423 (12)	0.0427 (12)	0.0422 (13)	−0.0001 (10)	0.0096 (10)	−0.0008 (9)
C7	0.0473 (13)	0.0368 (11)	0.0382 (12)	0.0033 (9)	0.0105 (9)	−0.0011 (9)
C8	0.0468 (12)	0.0344 (11)	0.0408 (12)	0.0001 (9)	0.0117 (10)	−0.0018 (9)
C9	0.0557 (14)	0.0454 (13)	0.0406 (13)	0.0001 (11)	0.0121 (11)	−0.0007 (10)
C10	0.0529 (15)	0.0711 (18)	0.0394 (13)	0.0053 (13)	0.0088 (11)	0.0095 (12)
C11	0.096 (3)	0.103 (3)	0.077 (2)	0.036 (2)	0.030 (2)	0.041 (2)
C12	0.096 (3)	0.201 (6)	0.088 (3)	0.062 (4)	0.027 (3)	0.080 (4)
C13	0.084 (3)	0.239 (7)	0.048 (2)	0.048 (4)	0.0111 (19)	0.009 (3)
C14	0.075 (2)	0.159 (4)	0.054 (2)	−0.002 (3)	0.0134 (18)	−0.026 (2)
C15	0.0710 (19)	0.082 (2)	0.0470 (16)	−0.0044 (16)	0.0143 (14)	−0.0112 (14)
C16	0.0493 (13)	0.0390 (12)	0.0583 (15)	−0.0051 (10)	0.0247 (11)	−0.0107 (10)
C17	0.0608 (17)	0.0495 (15)	0.089 (2)	−0.0012 (13)	0.0323 (16)	0.0023 (14)
C18	0.075 (2)	0.0549 (18)	0.111 (3)	−0.0139 (16)	0.048 (2)	0.0015 (17)
C19	0.0589 (18)	0.074 (2)	0.098 (3)	−0.0205 (16)	0.0417 (18)	−0.0206 (18)
C20	0.0502 (16)	0.070 (2)	0.103 (3)	0.0043 (15)	0.0174 (17)	−0.0066 (18)
C21	0.0603 (17)	0.0485 (16)	0.095 (2)	−0.0012 (13)	0.0212 (16)	0.0032 (15)
C22	0.0547 (14)	0.0403 (12)	0.0437 (13)	0.0073 (11)	0.0133 (11)	−0.0016 (10)
C23	0.0586 (15)	0.0385 (12)	0.0474 (13)	0.0084 (11)	0.0086 (11)	−0.0010 (10)
C24	0.0546 (14)	0.0399 (12)	0.0461 (13)	0.0059 (10)	0.0151 (11)	−0.0004 (10)
C25	0.0513 (13)	0.0323 (11)	0.0447 (13)	0.0053 (9)	0.0148 (10)	0.0043 (9)
C26	0.0495 (13)	0.0335 (11)	0.0502 (14)	0.0042 (10)	0.0150 (11)	0.0005 (9)
C27	0.0649 (17)	0.0376 (13)	0.0623 (17)	−0.0009 (11)	0.0185 (13)	−0.0036 (11)
C28	0.0675 (18)	0.0464 (15)	0.0667 (18)	−0.0111 (13)	0.0091 (14)	0.0007 (13)
C29	0.0491 (15)	0.0585 (17)	0.0708 (19)	−0.0045 (13)	0.0118 (13)	0.0112 (14)
C30	0.0554 (15)	0.0482 (14)	0.0582 (16)	0.0116 (12)	0.0181 (12)	0.0087 (12)
N1	0.0537 (12)	0.0294 (9)	0.0477 (11)	−0.0006 (8)	0.0162 (9)	−0.0028 (8)
N2	0.0545 (14)	0.0492 (13)	0.0818 (17)	0.0050 (11)	0.0230 (12)	−0.0124 (12)
O1	0.0597 (12)	0.0545 (11)	0.0533 (11)	0.0029 (9)	0.0005 (9)	0.0045 (8)
O2	0.0983 (18)	0.0445 (11)	0.0851 (16)	−0.0140 (11)	0.0261 (13)	−0.0162 (10)
O3	0.0870 (15)	0.0531 (11)	0.0750 (14)	−0.0218 (10)	0.0464 (12)	−0.0249 (10)
O4	0.0541 (11)	0.0398 (10)	0.1143 (18)	0.0102 (8)	0.0298 (11)	−0.0046 (10)
O5	0.0734 (13)	0.0507 (11)	0.0512 (11)	0.0185 (9)	0.0009 (9)	−0.0003 (8)
O6	0.0656 (14)	0.0686 (15)	0.141 (2)	−0.0149 (12)	0.0477 (15)	−0.0346 (15)
O7	0.0705 (16)	0.0616 (15)	0.209 (4)	0.0209 (13)	0.0318 (19)	−0.0169 (19)
S1	0.0583 (4)	0.0381 (3)	0.0422 (3)	−0.0027 (3)	0.0095 (3)	−0.0018 (2)
S2	0.0520 (4)	0.0350 (3)	0.0717 (5)	−0.0048 (3)	0.0303 (3)	−0.0119 (3)

### Geometric parameters (Å, °)

**Table:**

C1—C2	1.390 (3)	C17—C18	1.386 (4)
C1—C6	1.402 (3)	C17—H17	0.9300
C1—N1	1.413 (3)	C18—C19	1.370 (5)
C2—C3	1.393 (4)	C18—H18	0.9300
C2—H2	0.9300	C19—C20	1.353 (5)
C3—C4	1.384 (5)	C19—H19	0.9300
C3—H3	0.9300	C20—C21	1.383 (4)
C4—C5	1.370 (4)	C20—H20	0.9300
C4—H4	0.9300	C21—H21	0.9300
C5—C6	1.399 (4)	C22—O5	1.215 (3)
C5—H5	0.9300	C22—C23	1.473 (4)
C6—C7	1.442 (3)	C23—C24	1.329 (3)
C7—C8	1.352 (3)	C23—H23	0.9300
C7—C22	1.486 (3)	C24—C25	1.461 (3)
C8—N1	1.411 (3)	C24—H24	0.9300
C8—C9	1.489 (3)	C25—C30	1.397 (4)
C9—S1	1.792 (3)	C25—C26	1.404 (3)
C9—H9A	0.9700	C26—C27	1.369 (4)
C9—H9B	0.9700	C26—N2	1.458 (3)
C10—C11	1.364 (4)	C27—C28	1.367 (4)
C10—C15	1.398 (4)	C27—H27	0.9300
C10—S1	1.756 (3)	C28—C29	1.380 (4)
C11—C12	1.428 (7)	C28—H28	0.9300
C11—H11	0.9300	C29—C30	1.370 (4)
C12—C13	1.372 (8)	C29—H29	0.9300
C12—H12	0.9300	C30—H30	0.9300
C13—C14	1.335 (7)	N1—S2	1.6749 (19)
C13—H13	0.9300	N2—O6	1.211 (3)
C14—C15	1.369 (5)	N2—O7	1.212 (3)
C14—H14	0.9300	O1—S1	1.422 (2)
C15—H15	0.9300	O2—S1	1.424 (2)
C16—C17	1.368 (4)	O3—S2	1.414 (2)
C16—C21	1.371 (4)	O4—S2	1.423 (2)
C16—S2	1.759 (3)		
			
C2—C1—C6	122.0 (2)	C17—C18—H18	120.1
C2—C1—N1	130.7 (2)	C20—C19—C18	121.3 (3)
C6—C1—N1	107.3 (2)	C20—C19—H19	119.4
C1—C2—C3	116.6 (3)	C18—C19—H19	119.4
C1—C2—H2	121.7	C19—C20—C21	119.6 (3)
C3—C2—H2	121.7	C19—C20—H20	120.2
C4—C3—C2	122.1 (3)	C21—C20—H20	120.2
C4—C3—H3	119.0	C16—C21—C20	119.3 (3)
C2—C3—H3	119.0	C16—C21—H21	120.3
C5—C4—C3	121.0 (3)	C20—C21—H21	120.3
C5—C4—H4	119.5	O5—C22—C23	121.9 (2)
C3—C4—H4	119.5	O5—C22—C7	121.4 (2)
C4—C5—C6	118.8 (3)	C23—C22—C7	116.6 (2)
C4—C5—H5	120.6	C24—C23—C22	120.0 (2)
C6—C5—H5	120.6	C24—C23—H23	120.0
C5—C6—C1	119.6 (2)	C22—C23—H23	120.0
C5—C6—C7	133.2 (2)	C23—C24—C25	125.2 (2)
C1—C6—C7	107.1 (2)	C23—C24—H24	117.4
C8—C7—C6	108.6 (2)	C25—C24—H24	117.4
C8—C7—C22	124.6 (2)	C30—C25—C26	114.9 (2)
C6—C7—C22	126.8 (2)	C30—C25—C24	121.0 (2)
C7—C8—N1	108.7 (2)	C26—C25—C24	123.7 (2)
C7—C8—C9	126.9 (2)	C27—C26—C25	123.3 (2)
N1—C8—C9	124.3 (2)	C27—C26—N2	116.7 (2)
C8—C9—S1	112.79 (18)	C25—C26—N2	120.0 (2)
C8—C9—H9A	109.0	C28—C27—C26	119.7 (3)
S1—C9—H9A	109.0	C28—C27—H27	120.1
C8—C9—H9B	109.0	C26—C27—H27	120.1
S1—C9—H9B	109.0	C27—C28—C29	119.2 (3)
H9A—C9—H9B	107.8	C27—C28—H28	120.4
C11—C10—C15	121.9 (3)	C29—C28—H28	120.4
C11—C10—S1	120.7 (3)	C30—C29—C28	120.6 (3)
C15—C10—S1	117.4 (2)	C30—C29—H29	119.7
C10—C11—C12	117.0 (4)	C28—C29—H29	119.7
C10—C11—H11	121.5	C29—C30—C25	122.2 (3)
C12—C11—H11	121.5	C29—C30—H30	118.9
C13—C12—C11	119.4 (4)	C25—C30—H30	118.9
C13—C12—H12	120.3	C8—N1—C1	108.23 (18)
C11—C12—H12	120.3	C8—N1—S2	127.19 (17)
C14—C13—C12	122.4 (4)	C1—N1—S2	122.25 (16)
C14—C13—H13	118.8	O6—N2—O7	122.5 (3)
C12—C13—H13	118.8	O6—N2—C26	119.4 (2)
C13—C14—C15	119.9 (5)	O7—N2—C26	118.0 (2)
C13—C14—H14	120.1	O1—S1—O2	118.14 (14)
C15—C14—H14	120.1	O1—S1—C10	108.39 (13)
C14—C15—C10	119.4 (4)	O2—S1—C10	109.61 (15)
C14—C15—H15	120.3	O1—S1—C9	109.06 (12)
C10—C15—H15	120.3	O2—S1—C9	107.57 (14)
C17—C16—C21	121.5 (3)	C10—S1—C9	103.01 (13)
C17—C16—S2	118.4 (2)	O3—S2—O4	119.76 (14)
C21—C16—S2	120.0 (2)	O3—S2—N1	106.98 (11)
C16—C17—C18	118.6 (3)	O4—S2—N1	106.10 (12)
C16—C17—H17	120.7	O3—S2—C16	109.18 (13)
C18—C17—H17	120.7	O4—S2—C16	109.20 (13)
C19—C18—C17	119.8 (3)	N1—S2—C16	104.53 (11)
C19—C18—H18	120.1		
			
C6—C1—C2—C3	−0.4 (4)	C23—C24—C25—C26	155.7 (3)
N1—C1—C2—C3	179.2 (3)	C30—C25—C26—C27	−2.2 (4)
C1—C2—C3—C4	−0.8 (4)	C24—C25—C26—C27	171.1 (2)
C2—C3—C4—C5	0.9 (5)	C30—C25—C26—N2	175.7 (2)
C3—C4—C5—C6	0.3 (5)	C24—C25—C26—N2	−11.0 (4)
C4—C5—C6—C1	−1.4 (4)	C25—C26—C27—C28	2.3 (4)
C4—C5—C6—C7	−177.1 (3)	N2—C26—C27—C28	−175.7 (3)
C2—C1—C6—C5	1.5 (4)	C26—C27—C28—C29	−0.2 (4)
N1—C1—C6—C5	−178.2 (2)	C27—C28—C29—C30	−1.8 (4)
C2—C1—C6—C7	178.2 (2)	C28—C29—C30—C25	1.9 (4)
N1—C1—C6—C7	−1.5 (3)	C26—C25—C30—C29	0.1 (4)
C5—C6—C7—C8	175.8 (3)	C24—C25—C30—C29	−173.4 (3)
C1—C6—C7—C8	−0.3 (3)	C7—C8—N1—C1	−2.9 (3)
C5—C6—C7—C22	−4.2 (5)	C9—C8—N1—C1	−179.0 (2)
C1—C6—C7—C22	179.7 (2)	C7—C8—N1—S2	−165.70 (19)
C6—C7—C8—N1	2.0 (3)	C9—C8—N1—S2	18.2 (3)
C22—C7—C8—N1	−178.1 (2)	C2—C1—N1—C8	−177.0 (3)
C6—C7—C8—C9	178.0 (2)	C6—C1—N1—C8	2.7 (3)
C22—C7—C8—C9	−2.1 (4)	C2—C1—N1—S2	−13.2 (4)
C7—C8—C9—S1	−88.4 (3)	C6—C1—N1—S2	166.49 (17)
N1—C8—C9—S1	87.1 (3)	C27—C26—N2—O6	150.7 (3)
C15—C10—C11—C12	0.0 (6)	C25—C26—N2—O6	−27.4 (4)
S1—C10—C11—C12	179.5 (3)	C27—C26—N2—O7	−25.9 (4)
C10—C11—C12—C13	1.5 (7)	C25—C26—N2—O7	156.1 (3)
C11—C12—C13—C14	−2.3 (8)	C11—C10—S1—O1	149.5 (3)
C12—C13—C14—C15	1.5 (8)	C15—C10—S1—O1	−30.9 (3)
C13—C14—C15—C10	0.0 (6)	C11—C10—S1—O2	19.3 (3)
C11—C10—C15—C14	−0.8 (5)	C15—C10—S1—O2	−161.1 (2)
S1—C10—C15—C14	179.7 (3)	C11—C10—S1—C9	−95.0 (3)
C21—C16—C17—C18	−1.2 (5)	C15—C10—S1—C9	84.6 (3)
S2—C16—C17—C18	174.7 (3)	C8—C9—S1—O1	−50.7 (2)
C16—C17—C18—C19	1.6 (5)	C8—C9—S1—O2	78.5 (2)
C17—C18—C19—C20	−0.3 (6)	C8—C9—S1—C10	−165.71 (19)
C18—C19—C20—C21	−1.4 (6)	C8—N1—S2—O3	−28.1 (2)
C17—C16—C21—C20	−0.5 (5)	C1—N1—S2—O3	171.30 (19)
S2—C16—C21—C20	−176.3 (3)	C8—N1—S2—O4	−157.0 (2)
C19—C20—C21—C16	1.8 (5)	C1—N1—S2—O4	42.4 (2)
C8—C7—C22—O5	−37.3 (4)	C8—N1—S2—C16	87.6 (2)
C6—C7—C22—O5	142.6 (3)	C1—N1—S2—C16	−73.0 (2)
C8—C7—C22—C23	141.9 (3)	C17—C16—S2—O3	−121.2 (2)
C6—C7—C22—C23	−38.1 (4)	C21—C16—S2—O3	54.7 (3)
O5—C22—C23—C24	−18.1 (4)	C17—C16—S2—O4	11.4 (3)
C7—C22—C23—C24	162.6 (2)	C21—C16—S2—O4	−172.6 (2)
C22—C23—C24—C25	173.4 (2)	C17—C16—S2—N1	124.6 (2)
C23—C24—C25—C30	−31.4 (4)	C21—C16—S2—N1	−59.4 (3)

### Hydrogen-bond geometry (Å, °)

**Table:**

*D*—H···*A*	*D*—H	H···*A*	*D*···*A*	*D*—H···*A*
C27—H27···O2^i^	0.93	2.56	3.221 (3)	128.
C13—H13···O1^ii^	0.93	2.58	3.275 (5)	132.
C19—H19···O4^iii^	0.93	2.58	3.222 (4)	126.
C2—H2···O4i	0.93	2.37	2.946 (4)	120.
C9—H9A···O3i	0.97	2.21	2.846 (3)	122.
C9—H9B···O5i	0.97	2.37	3.029 (3)	125.

Symmetry codes: (i) −*x*+1, −*y*, −*z*; (ii) *x*+1/2, −*y*+1/2, *z*+1/2; (iii) *x*+1, *y*, *z*; i.
